# Supplementary material for: Genome-wide CRISPR Screens in T Helper Cells Reveal Pervasive Crosstalk between Activation and Differentiation
Source: Cell. 2019 Feb 7;176(4):882–896.e18. doi: 10.1016/j.cell.2018.11.044 (PMC6370901; doi:10.1016/j.cell.2018.11.044)
Supplement: Data S2. Processed Data from All the Steps of the Analysis, Related to Figure 1 [file mmc2.zip › supplemental data/motif analysis/Bhlhe40_homer/homerResults/motif4.similar.html]

motif4

## Information for motif4

C
T
G
A
G
A
T
C
G
A
C
T
A
C
G
T
A
G
T
C
G
A
T
C
C
G
A
T
A
C
T
G
A
G
C
T
C
A
G
T
  
Reverse Opposite:  

G
T
C
A
T
C
G
A
T
G
A
C
G
C
T
A
C
T
A
G
T
C
A
G
T
G
C
A
C
T
G
A
C
T
A
G
G
A
C
T
  

|  |  |
| --- | --- |
| p-value: | 1e-23 |
| log p-value: | -5.423e+01 |
| Information Content per bp: | 1.479 |
| Number of Target Sequences with motif | 158.0 |
| Percentage of Target Sequences with motif | 13.17% |
| Number of Background Sequences with motif | 2302.1 |
| Percentage of Background Sequences with motif | 5.41% |
| Average Position of motif in Targets | 108.3 +/- 54.2bp |
| Average Position of motif in Background | 98.1 +/- 62.0bp |
| Strand Bias (log2 ratio + to - strand density) | 0.2 |
| Multiplicity (# of sites on avg that occur together) | 1.11 |
| Motif File: | file (matrix) reverse opposite |

### Similar de novo motifs found

|  |  |  |  |  |  |  |  |
| --- | --- | --- | --- | --- | --- | --- | --- |
| Rank | Match Score | Redundant Motif | P-value | log P-value | % of Targets | % of Background | Motif file |
| 1 | 0.856 | G T C A A C T G T A C G C G T A C G T A C T A G A C G T T C A G | 1e-15 | -36.171488 | 18.75% | 10.77% | motif file (matrix) |
| 2 | 0.615 | T C A G A G C T C G A T A G C T T G A C A G T C A C T G T A C G | 1e-13 | -32.022064 | 14.42% | 7.85% | motif file (matrix) |
| 3 | 0.625 | T A C G A G C T T C G A T C G A T G A C T A G C C A T G A C T G T C G A G C T A | 1e-12 | -28.203683 | 7.83% | 3.46% | motif file (matrix) |
| 4 | 0.776 | A G C T A G C T G A T C G T C A A G C T A G C T C A G T A G T C G T A C C G A T A T C G A G C T | 1e-11 | -26.288057 | 12.58% | 7.00% | motif file (matrix) |
